# Efecto del enjuague bucal con cloruro de cetilpiridinio en la microdureza superficial del polimetilmetacrilato de autocurado, termocurado y CAD CAM. Estudio *in vitro*

**DOI:** 10.21142/2523-2754-1301-2025-227

**Published:** 2025-03-03

**Authors:** Karol Milagros Meza Zegarra, Ana Isabel López Flores

**Affiliations:** 1 Carrera de Estomatologia, Universidad Cientifica del Sur. Lima, Peru. karol.meza.zegarra.91@gmail.com Universidad Científica del Sur Carrera de Estomatologia Universidad Cientifica del Sur Lima Peru karol.meza.zegarra.91@gmail.com; 2 Departamento de Posgrado, Carrera de Estomatologia, Universidad Cientifica del Sur. Lima, Peru. dra.lopz@gmail.com Universidad Científica del Sur Departamento de Posgrado Carrera de Estomatologia Universidad Cientifica del Sur Lima Peru dra.lopz@gmail.com

**Keywords:** PMMA, antisépticos bucales, dureza, compuesto de amonio cuaternario (DeCS), PMMA, oral antiseptics, hardness, quaternary ammonium compound (DeCS)

## Abstract

**Objetivo:**

Evaluar el efecto del enjuague bucal Vitis CPC Protect en la microdureza superficial de tres tipos de polimetilmetacrilato (PMMA): autocurado (Alike), termocurado (Vitalloy) y CAD/CAM (Telio CAD).

**Metodología:**

Se fabricaron 90 discos de PMMA, divididos en tres grupos de 30 discos, con dimensiones de 2 mm de espesor y 10 mm de diámetro. Cada grupo se subdividió en tres subgrupos (n = 10): el primero para medir la microdureza inicial, el segundo se sumergió en agua destilada como control, y el tercero en enjuague bucal Vitis CPC Protect. Los discos se mantuvieron en soluciones de 20 ml durante 6 horas a 37 °C, para simular el uso diario del enjuague dos veces al día durante 6 meses. La microdureza superficial Vickers se midió antes y después de la inmersión utilizando un durómetro Vickers con una carga de 50 g. Posteriormente, los datos fueron analizados estadísticamente mediante el test Anova de un factor y el test de comparaciones múltiples deTukey.

**Resultados:**

Los valores iniciales de microdureza fueron los siguientes: 14,05 ± 0,31 para el autocurado, 18,00 ± 0,60 para el termocurado y 20,35 ± 1,80 para CAD/CAM. Después de la inmersión en el enjuague bucal, los valores fueron los siguientes: 13,81 ± 0,20 para el autocurado, 17,20 ± 0,38 para el termocurado y 19,21 ± 0,60 para CAD/CAM. Los análisis Anova y la prueba de Tukey revelaron diferencias estadísticamente significativas en la microdureza de los PMMA termocurado y CAD/CAM (p < 0.05).

**Conclusión:**

El enjuague bucal con cloruro de cetilpiridinio afecta la microdureza superficial de los PMMA de termocurado y CAD/CAM, siendo este último el más resistente a dicho efecto. Sin embargo, al ser la disminución mínima, no se contraindicaría el uso del enjuague bucal en los pacientes con restauraciones provisionales de PMMA.

## Abstract (en)

**Objective:**

To evaluate the effect of Vitis CPC Protect mouthwash on the surface microhardness of three types of polymethylmethacrylate (PMMA): self-curing (Alike), thermocuring (Vitalloy) and CAD/CAM (Telium CAD)**.**

**Methodology:**

90 PMMA discs were manufactured, divided into three groups of 30 discs, with dimensions of 2 mm thick and 10 mm in diameter. Each group was subdivided into three subgroups (n = 10): The first subgroup to measure baseline microhardness, the second was immersed in distilled water as a control, and the third in Vitis CPC Protect mouthwash. The discs were kept in 20 ml solutions for 6 hours at 37°C, simulating daily use of rinsing twice a day for 6 months. Vickers surface microhardness was measured before and after immersion using a Vickers durometer with a load of 50 g. Subsequently, the data were statistically analyzed using the One-Way ANOVA test and Tukey's Multiple Comparisons test.

**Results:**

The initial microhardness values were: self-curing 14.05 ± 0.31, thermocuring 18.00 ± 0.60, and CAD/CAM 20.35 ± 1.80. After immersion in mouthwash, the values were: self-curing 13.81 ± 0.20, thermocuring 17.20 ± 0.38, and CAD/CAM 19.21 ± 0.60. ANOVA analyses and Tukey's test revealed statistically significant differences in the microhardness of thermocured PMMA and CAD/CAM (p < 0.05).

**Conclusion:**

The mouthwash with cetylpyridinium chloride affects the surface microhardness of thermally polymerized and CAD/CAM PMMA, with the latter being the more resistant to these effects. However, since the decrease is minimal, the use of mouthwash would not be contraindicated for patients with provisional PMMA restorations.

## INTRODUCCIÓN

Las restauraciones provisionales son esenciales para el éxito de las definitivas, pero deben cumplir requisitos biológicos, biomecánicos y estéticos. Es necesario que sean biocompatibles, no irritantes y ofrezcan una superficie pulida. Además, tienen que ser fuertes y duraderas, para resistir las fuerzas de masticación ^1^. Pueden confeccionarse de forma directa o indirecta, dependiendo de factores como el tiempo de trabajo, la facilidad de manejo y el costo ^2^.

El polimetilmetacrilato (PMMA) se usa comúnmente para estas restauraciones y puede polimerizarse por calor (termocurado) o químicamente (autocurado) ^3^. Sin embargo, el autocurado tiene un menor grado de polimerización y mayor cantidad de monómeros residuales; asimismo, el calor resultante de la polimerización exotérmica puede causar necrosis pulpar en un alto porcentaje de casos ^4^. El PMMA de termocurado incluye menos monómeros residuales y es menos citotóxico ^5^^,^^6^.

Existen también los bloques de PMMA CAD/CAM para la confección de restauraciones provisionales; estos son polimerizados industrialmente a alta temperatura y presión para evitar porosidad. El resultado es un producto con cadenas poliméricas muy largas, bajo contenido residual de monómeros y alta dureza ^7^. Aunque la química del PMMA CAD/CAM es similar a la del PMMA de termocurado, el PMMA CAD/CAM muestra superioridad en muchas propiedades, incluida la dureza. Ambos materiales son igualmente biocompatibles y sin diferencias significativas en términos de liberación de monómeros ^8^.

Para mantener cualquier tipo de prótesis en boca es importante la salud bucal, y el uso de enjuagues bucales se recomienda como complemento del cepillado dental por su efecto refrescante y antiséptico ^9^. La falta de higiene puede aumentar la rugosidad del material y el desarrollo de caries secundarias alrededor de las restauraciones ^10^^,^^11^. En ese sentido, los PMMA deben soportar los diferentes estímulos que pueden afectar su composición, aunque presentan ciertas desventajas, ya que se hinchan y disuelven en muchos disolventes orgánicos y productos químicos, debido a sus grupos de ésteres fácilmente hidrolizables ^12^. Por ello, el estar en contacto diario con enjuagues bucales podría alterar su dureza ^9^^,^^13^^,^^14^.

La dureza es una propiedad física de gran importancia, pues representa el grado de solidez producida por la cohesión entre partículas que componen una sustancia y se define como la resistencia de un material al corte o indentación. Se relaciona con la resistencia a la compresión, al ablandamiento intraoral y el grado de conversión ^15^. Un bajo valor de dureza de la superficie se relaciona, en gran medida, con la resistencia inadecuada al desgaste ^16^ y la tendencia al raspado, lo que puede fatigar el material y conducir a la falla de la restauración ^17^. Una de las pruebas que más se utiliza para la medición de dureza en materiales dentales es la prueba de Vickers Hardness, mediante la penetración de un diamante con forma piramidal sobre la superficie de la muestra ^15^.

Estudios previos han demostrado que los enjuagues bucales con base en aceites esenciales afectan la microdureza de los PMMA de autocurado y termocurado, y que este último es más resistente tras 270 minutos de inmersión con termociclaje ^18^. Un estudio sobre enjuagues bucales con peróxido de hidrógeno, alcohol y flúor en cerámicas no mostró diferencias significativas en la microdureza ^19^. Sin embargo, otro estudio indicó que el enjuague bucal con flúor, al combinarse con los ácidos gástrico y acético, afecta significativamente la microdureza de las cerámicas CAD/CAM ^15^.

Hasta ahora, no se ha investigado el impacto del cloruro de cetilpiridinio (CPC) en estos materiales. El enjuague bucal con CPC al 0,07% es un amonio catiónico seguro para uso humano, con propiedades bactericidas y antivirales, incluidos el virus de la influenza y el coronavirus ^20^. Dado que los enjuagues bucales son frecuentemente recomendados durante la fase de provisionalización, este estudio evaluó el efecto del enjuague con CPC en la microdureza superficial de tres tipos de PMMA durante un período simulado de seis meses.

## MATERIALES Y MÉTODOS

En este estudio experimental in vitro, con código de registro N.° POS-115-2022-00361, aprobado mediante la revisión por pares y exonerado del pase a Comité de Ética de la Universidad Científica del Sur (CIEI Científica), se seleccionaron tres tipos de polimetilmetacrilato (PMMA): PMMA de autocurado, PMMA de termocurado y PMMA para uso en CAD/CAM, así como el enjuague bucal Vitis CPC Protect al 0,07%. Las marcas y composiciones químicas se encuentran en la Tabla 1 ^21^^-^^23^.

**Tabla 1:** Características de los materiales utilizados en el estudio

MATERIAL	MARCA	COMPOSICIÓN
Enjuague bucal	Vitis CPC Protect	Cloruro de cetilpiridinio (CPC) al 0,07%
PMMA autocurado	Alike	Líquido:
		Metacrilato de metilo: 75 - < 100% Metanol: 5 - < 10% Dimetacrilato**: 1 - < 2,5% Acelerante**: 1 - < 2,5% Absorbente de luz UV**: 1 - < 2,5%
		Polvo:
		Ftalato de dibutilo: 2,5 - < 5% Polimetacrilato de metilo: 2,5 - < 5% Peróxido de dibenzoilo: 0,5 - < 1%
PMMA termocurado	Vitalloy	No especificado.
PMMA CAD/CAM	Telio CAD	Polimetilmetacrilato (PMMA), pigmentos

Se fabricaron un total de 90 discos de PMMA, con 30 discos de cada tipo, según las especificaciones del fabricante. Las dimensiones fueron de 10 mm de diámetro y 2 mm de espesor. Cada grupo de 30 discos se dividió en tres subgrupos (n = 10), de acuerdo con la microdureza inicial, la inmersión en agua destilada o en enjuague bucal. 

Los criterios de selección incluyeron discos de PMMA de autocurado, termocurado y CAD CAM que presentaban dimensiones correctas y estaban correctamente pulidos. Se excluyeron aquellos que presentaban fisuras, burbujas o cualquier alteración en su superficie, así como los que no cumplían las dimensiones especificadas.

Para confeccionar los discos del grupo 1, se utilizó acrílico de autopolimerización (Alike), mezclando con una espátula de cemento el polímero y el monómero en una relación de 3 a 1 durante 10 a 15 segundos. Se aplicó vaselina como lubricante a las matrices metálicas fabricadas de acuerdo a las dimensiones, se vertió el acrílico y se cubrió con otra platina hasta alcanzar su polimerización. Una vez polimerizados, los discos se desmoldaron, se retiraron los excesos con una fresa de tungsteno y se pulieron con puntas de silicona. Finalmente, se almacenaron en una cubeta plástica negra protegida de la luz y rotulada.

La confección de los discos del grupo 2 fue realizada en el laboratorio dental Silident, de la ciudad de Arequipa (Perú), siguiendo los protocolos conocidos para este tipo de material de polimerización térmica ^16^. Una vez obtenidas las muestras, se procedió a retirar los excesos con un pimpollo de tungsteno y se pulieron las superficies con puntas de silicona. Los discos fueron almacenados en una cubeta plástica negra protegida de la luz.

Las muestras fresadas del tercer grupo se fabricaron en el laboratorio Jusephy (Arequipa, Perú) utilizando la tecnología CAD/CAM (diseño asistido por computadora/fabricación asistida por computadora). El disco fue diseñado utilizando el *software* CAD Tinkercad (Autodesk, San Rafael, CA, EE. UU.), con dimensiones específicas de 10 mm de diámetro y 2 mm de espesor. El diseño fue exportado y enviado al *software* inLab CAM SW 22.3 (Dentsply Sirona, York, PA, EE. UU.), que es el encargado de enviar las órdenes a la fresadora, en este caso la CAM MCX5 (Dentsply Sirona, York, PA, EE. UU.). El disco utilizado para el fresado ya viene preformado de fábrica. Las superficies se pulieron con puntas de silicona y se almacenaron en una cubeta plástica negra (figura 3).

La inmersión de los discos se realizó en agua destilada (control) con el enjuague bucal Vitis CPC Protect (experimental), en cada subgrupo de acuerdo con el tipo de PMMA. Estas muestras permanecieron en sus cubetas plásticas negras, rotuladas y protegidas de la luz con 20 ml de las soluciones mencionadas a 37 °C durante 6 horas, lo que equivale a 2 minutos de uso diario del enjuague bucal durante 6 meses, tal como lo describen El-Badrawy, McComb Wood (1993) y Wistrom, Diaz-Arnold Swift (1994) ^19^^,^^24^. Después del periodo de inmersión, los discos se lavaron con agua corriente durante 20 segundos y se secaron con toallas de papel, para después ser sometidas a la prueba de microdureza Vickers.

Estas pruebas se realizaron en el laboratorio High Technology Laboratory Certificate utilizando un equipo de microdureza Vickers marca LG, modelo HV1000, que mide automáticamente las huellas dejadas por el penetrador, La dureza Vickers (HV) se calcula usando la fórmula correspondiente: HV = 1.854 * (P / d²), donde HV es el número de dureza en kg/mm², P es la carga indentadora en kg y d es la longitud diagonal de la impresión, teniendo en cuenta la norma ISO 6507.

**Figura 1:**
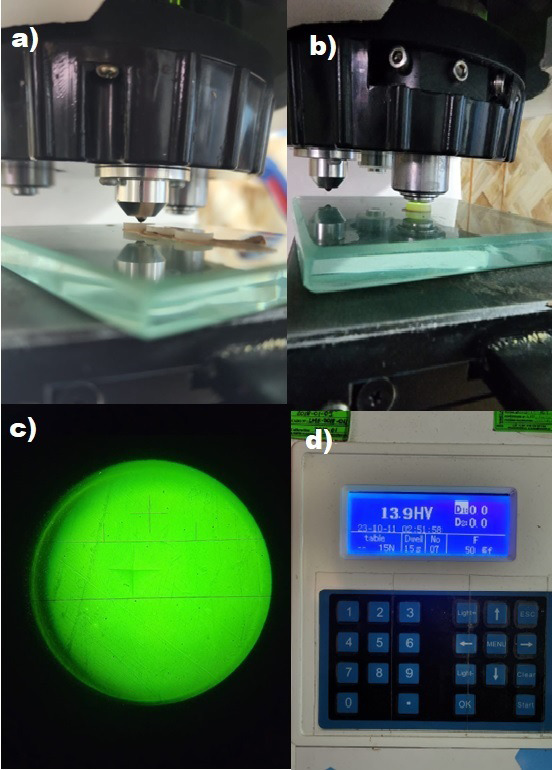
a) Penetración del microidentador a la muestra. b) Evaluación microscópica de la microidentación. c) Medición de diagonales. d) Valor de la microdureza Vickers.

Se realizaron 3 indentaciones en la superficie de cada disco, cuyo promedio se consideró como el valor de microdureza por disco, bajo una carga de 50 g durante 15 segundos, con hendiduras a 500 μm de distancia entre sí. Con el lente óptico de 50x, se midieron las dos longitudes diagonales de cada hendidura y los datos se ingresaron al equipo para calcular la microdureza al inicio y después de ser sumergidas las muestras en las dos soluciones (Figura 1).

### Análisis estadístico

Los valores de microdureza superficial obtenidos antes y después de la inmersión se analizaron estadísticamente para determinar si existían diferencias significativas entre los subgrupos. Se utilizó un análisis de varianza (Anova) seguido de la prueba de comparaciones múltiples de Tukey para identificar diferencias significativas entre las medias de los grupos. Se utilizó el *software* SPSS versión 26 (IBM, EE. UU.).

## RESULTADOS

Después de realizar el ensayo de microdureza superficial inicial de los tres tipos de PMMA como valores base, el valor promedio fue de 14,05 ± 0,31 para el autocurado, 18,00 ± 0,60 para el termocurado y de 20,35 ± 1,80 para CAD CAM (Tabla 2).

**Tabla 2:** Comparación de la microdureza superficial inicial de los polometilmetacrilatos de autocurado, termocurado y CAD CAM

PMMA	n	Media	DE	Mín.	Máx.	p1	p2
1. Autocurado	10	14,05	0,31	13,7	14,8	<0,001	<0,001 entre 1 y 2
2. Termocurado	10	18,00	0,60	17,3	19,1		<0,001 entre 1 y 3
3. CAD CAM	10	20,35	1,80	17,8	23,4		<0,001 entre 2 y 3

p1 Anova de un Factor; p2 Prueba HSD de Tukey

La comparación estadística mediante Anova de un factor mostró diferencias entre los grupos (p < 0,001). Además, las pruebas HSD de Tukey indicaron diferencias significativas en la microdureza entre los tres tipos de PMMA (p < 0,001).

Los resultados obtenidos para los segundos subgrupos de cada tipo de PMMA en la medición de la microdureza superficial, después de ser sumergidos en agua destilada como grupo control, fueron los siguientes valores promedio: 13,55 ± 0,39 para autocurado, 17,21 ± 0,86 para termocurado y 18,84 ± 0,67 para CAD/CAM.

La comparación estadística mediante Anova de un factor mostró diferencias significativas entre los grupos (p < 0,001). Las pruebas HSD de Tukey indicaron diferencias significativas en la microdureza entre el PMMA de autocurado y termocurado (p < 0,001), entre el PMMA de termocurado y CAD CAM (p < 0,001), y entre el PMMA de autocurado y CAD CAM (p < 0,001).

Para el tercer subgrupo, que corresponde a los discos sumergidos en el enjuague bucal Vitis CPC Protect de cada tipo de PMMA, los valores promedio obtenidos fueron los siguientes: 13,81 ± 0,20 para autocurado, 17,20 ± 0,38 para termocurado y 19,21 ± 0,60 para CAD/CAM (Tabla 3).

**Tabla 3:** Comparación de la microdureza superficial después de la inmersión de los PMMA en agua destilada y enjuague bucal

Estímulo	Grupo	n	Media	DE	Mín.	Máx.	p1	p2
Agua destilada	1. A	10	13,55	0,39	13,2	14,2	<0,001	<0,001 entre 1 y 2
	2. T	10	17,21	0,86	16,2	18,7		<0,001 entre 1 y 3
	3. C	10	18,84	0,67	18,0	19,9		<0,001 entre 2 y 3
CPC Vitis	1. A	10	13,81	0,20	13,5	14,2	<0,001	<0,001 entre 1 y 2
	2. T	10	17,20	0,38	16,5	17,7		<0,001 entre 1 y 3
	3. C	10	19,21	0,60	18,5	20,6		<0,001 entre 2 y 3

p1 Anova de un factor; p2 prueba HSD de Tukey

A: Autocurado. T: Termocurado. C: CAD CAM

La comparación estadística mediante Anova de un factor mostró diferencias significativas entre los grupos (p < 0,001). Las pruebas HSD de Tukey indicaron diferencias significativas en la microdureza entre los tres tipos de PMMA (p < 0,001).

Para el PMMA de autocurado, la microdureza inicial fue de 14,05 ± 0,31. Después de la exposición al enjuague bucal, disminuyó a 13,81 ± 0,20. Tras la exposición a agua destilada, fue aún menor, con un promedio de 13,55 ± 0. 

Para el PMMA de termocurado, la microdureza inicial fue de 18,00 ± 0,60. Después de la exposición al enjuague bucal, disminuyó a 17,20 ± 0,38. Tras la exposición a agua destilada, se mantuvo similar a 17,21 ± 0,86.

Para el PMMA CAD CAM, la microdureza inicial fue de 20,35 ± 1,80. Después de la exposición al enjuague bucal, disminuyó a 19,21 ± 0,60. Tras la exposición a agua destilada, fue de 18,84 ± 0 (Tabla 4).

**Tabla 4:** Comparación de la microdureza superficial de los tres tipos de PMMA según la inmersión

Grupo	Medición	n	Media	DE	Mín.	Máx.	p1	p2
Autocurado	1. MI	10	14,05	0,31	13,7	14,8	<0,01	≥0,05 entre 1 y 2
	2. EV	10	13,81	0,20	13,5	14,2		<0,01 entre 1 y 3
	3. AD	10	13,55	0,39	13,2	14,2		<0,01 entre 2 y 3
Termocurado	1. MI	10	18,00	0,60	17,3	19,1	<0,01	<0,01 entre 1 y 2
	2. EV	10	17,20	0,38	16,5	17,7		<0,01 entre 1 y 3
	3. AD	10	17,21	0,86	16,2	18,7		≥0,05 entre 2 y 3
CAD CAM	1. MI	10	20,35	1,80	17,8	23,4	<0,01	<0,01 entre 1 y 2
	2. EV	10	19,21	0,60	18,5	20,6		<0,01 entre 1 y 3
	3. AD	10	18,84	0,67	18,0	19,9		≥0,05 entre 2 y 3

MI: microdureza inicial, EV: enjuague Vitis CPC Protect, AD: agua destilada

p1 Anova de un factor; p2 prueba HSD de Tukey

## DISCUSIÓN

El objetivo de este estudio fue evaluar el efecto del enjuague bucal con cloruro de cetilpiridinio en la microdureza superficial de los PMMA de autocurado, termocurado y CAD/CAM. Los valores iniciales de microdureza mostraron diferencias estadísticamente significativas entre los tres tipos de PMMA, lo que confirma las características de cada material según su proceso de fabricación y grado de polimerización ^4^^,^^7^^,^^8^.

En los resultados del PMMA de autocurado, se observó que no hubo una disminución estadísticamente significativa en su microdureza tras 6 meses de exposición al enjuague bucal, aunque este material presenta limitaciones para un uso prolongado ^4^. Su menor microdureza y mayor capacidad de absorción de agua lo hacen vulnerable a fallas bajo cargas cíclicas ^5^, lo que compromete su rendimiento mecánico en clínica.

Los resultados podrían deberse a la metodología del estudio, por lo que se sugiere incluir termociclado en futuras investigaciones para simular condiciones bucales y evaluar mejor la resistencia del material. Además, la reacción exotérmica del PMMA de autocurado podría si no es correctamente controlada ^4^, y su uso se ha limitado a reparaciones de prótesis dentales, lo que genera preocupación por mejorar las propiedades mecánicas de los materiales de reparación ^25^. En comparación con los otros tipos, el PMMA de autocurado plantea dudas sobre su aplicación.

Por otro lado, el PMMA de termocurado mostró una disminución estadísticamente significativa en su microdureza tras la inmersión en el enjuague bucal. Sin embargo, la microdureza de este material sigue siendo mayor que la del PMMA de autocurado, probablemente debido a su menor solubilidad y sorción ^26^. Aunque la reducción observada en un periodo equivalente a seis meses de uso no sería clínicamente relevante para contraindicar el uso de enjuague con cloruro de cetilpiridinio, sería importante continuar investigando su efecto a largo plazo.

En el caso del PMMA CAD/CAM, se registró una disminución estadísticamente significativa de 1,14 VHN en la microdureza tras la inmersión en el enjuague bucal. Sin embargo, este material mantiene la mayor microdureza entre los tres tipos de PMMA, lo que refuerza su superioridad en términos de resistencia a la degradación. Su proceso de polimerización industrial contribuye a mejorar sus propiedades mecánicas ^7^^,^^8^. A pesar de la disminución observada, no se considera clínicamente significativa en un periodo de 6 meses, considerando que este material puede permanecer en boca hasta por un año ^23^. Por lo tanto, no se justifica contraindicar el uso de enjuagues bucales con cloruro de cetilpiridinio.

Los hallazgos indican que la disminución de la microdureza de los PMMA tras la exposición al enjuague bucal es mínima y clínicamente irrelevante. Sin embargo, factores clínicos como las fuerzas mecánicas durante la masticación y la saliva, que contiene enzimas que pueden acelerar la degradación de las resinas acrílicas ^27^^,^^28^, también influyen en la dureza del material. La interacción entre la saliva y los productos de higiene bucal podría aumentar la pérdida de microdureza.

Además, los jugos gástricos, como se observa en estudios en cerámicas, confirman la disminución de dureza en materiales definitivos, lo que podría afectar aún más a los materiales provisionales. Se ha notado la falta de estudios sobre la influencia de los enjuagues bucales en estos materiales, lo que sugiere la necesidad de futuras investigaciones sobre los factores que alteran la microdureza.

Finalmente, los resultados apoyan el uso de PMMA CAD/CAM para restauraciones provisionales de mayor duración, mientras que el PMMA de termocurado también muestra un buen rendimiento. Se recomienda limitar el uso del PMMA de autocurado debido a sus propiedades mecánicas inferiores y mayor susceptibilidad a la degradación.

### Limitaciones

La presente investigación tuvo algunas limitaciones. En primer lugar, se enfocó únicamente en una sola marca de cada tipo de polimetilmetacrilato. Sin embargo, se evaluó las de uso más frecuente en la práctica clínica. Por otro lado, al existir más estudios sobre materiales definitivos y pocos sobre materiales para la confección de restauraciones provisionales, no hubo cómo contrastar los resultados con los de otros estudios. Pero nuestros resultados coinciden en que el uso de enjuagues bucales afecta la microdureza superficial de los materiales de restauración. Se recomienda más estudios sobre el tema para ser comparados con los resultados presentes.

## CONCLUSIÓN

El enjuague bucal con cloruro de cetilpiridinio afecta significativamente la microdureza superficial de los PMMA de termocurado y CAD/CAM, siendo este último el más resistente a dichos efectos.

## Financiamiento:

Autofinanciado
